# SVAMP: sequence variation analysis, maps and phylogeny

**DOI:** 10.1093/bioinformatics/btu176

**Published:** 2014-04-03

**Authors:** Raeece Naeem, Lailatul Hidayah, Mark D. Preston, Taane G. Clark, Arnab Pain

**Affiliations:** ^1^Pathogen Genomics Laboratory, Computational Bioscience Research Center, King Abdullah University of Science and Technology (KAUST), Thuwal, 23955-6900, Kingdom of Saudi Arabia and ^2^Department of Pathogen Molecular Biology, London School of Hygiene and Tropical Medicine, London WC1E 7HT, UK

## Abstract

**Summary:** SVAMP is a stand-alone desktop application to visualize genomic variants (in variant call format) in the context of geographical metadata. Users of SVAMP are able to generate phylogenetic trees and perform principal coordinate analysis in real time from variant call format (VCF) and associated metadata files. Allele frequency map, geographical map of isolates, *Tajima’s D* metric, single nucleotide polymorphism density, GC and variation density are also available for visualization in real time. We demonstrate the utility of SVAMP in tracking a methicillin-resistant *Staphylococcus aureus* outbreak from published next-generation sequencing data across 15 countries. We also demonstrate the scalability and accuracy of our software on 245 *Plasmodium falciparum* malaria isolates from three continents.

**Availability and implementation:** The Qt/C++ software code, binaries, user manual and example datasets are available at http://cbrc.kaust.edu.sa/svamp

**Contact:** arnab.pain@kaust.edu.sa or arnab.pain@cantab.net

**Supplementary information:** Supplementary data are available at *Bioinformatics* online.

## 1 INTRODUCTION

Associating sequence variants [single nucleotide polymorphisms (SNPs) and indels] with sample metadata such as geographical location and drug susceptibility have played a key role in studying the population structure (Manske *et al.*, 2012), identifying mechanisms of drug resistance (Downing *et al.*, 2011) and tracking the transmission of an infectious disease (Harris *et al.*, 2010). With the increasing application of deep sequencing as an approach, the number and volume of population studies with geo-biological information and associated genomic data will continue to grow. This increases the demand for tools to integrate, visualize and analyse complex genomic epidemiological data in real time, including browsing genome variation patterns and assessing population structure or geo-phylogeny. Although software such as *Polylens* (Berry *et al.*, 2013) and *GenGIS* (Parks *et al.*, 2009) can integrate geographical and genetic sequence data, there is a need to scale up to whole genome variation in the standardized VCF format (Danecek *et al.*, 2011) with informative population genetic analysis. This motivated us to develop SVAMP, a stand-alone Qt/C++ application capable of analysing variants in the context of geography and aiding in making inferences on the population structure. SVAMP is built on the open-source software *VarB* (Preston *et al.*, 2012).

## 2 METHODS

Input to SVAMP software is a bundle of multisample VCF file, reference FASTA, annotation general feature format (GFF) and a precalculated SQLite database file. The bundle preparation script included as a part of SVAMP software captures the geographical coordinates, date of isolation and the genome coverage of samples. The files when loaded into SVAMP will aid the user in performing key population genomics analysis in real time and visualize the results. Two popular methods of analysing sample relatedness, principal coordinate analysis [PCoA; Torgerson–Gower scaling (Gower, 1966)] and geo-phylogenetic tree, are integrated into SVAMP. The pairwise dissimilarity matrix D is first computed based on the Hamming distance (Hamming, 1950) (*d*) between pairs of samples (*i, j*) using equation

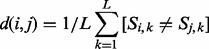

where *k* is the index of the genomic position out of L considered positions. S_i,k_ is the genotype called by sample *i* at position *k* in the genome. Positions that have missing genotype information are ignored in the computation; therefore, the multisample VCF file should ideally consist of samples and variants with reasonably complete data. The matrix D forms the basis for subsequent PCoA and phylogenetic tree reconstruction and consists of N (number of samples) rows and K (number of variant positions) columns.

**Fig. 1.**
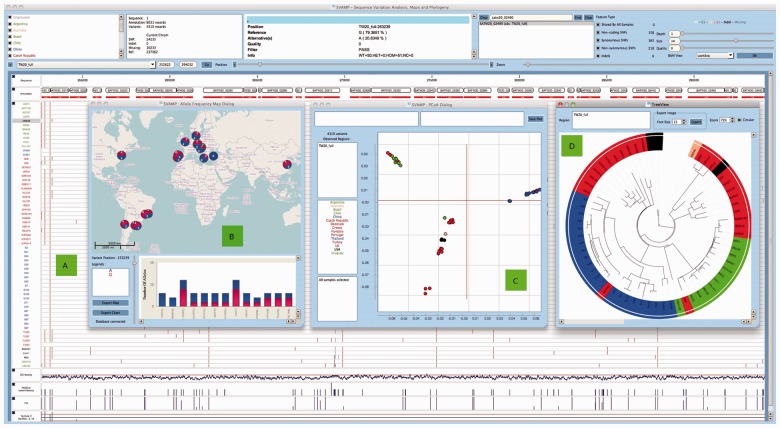
Screenshot from SVAMP software shows (**A**) variation across 63 MRSA isolates from 15 countries, (**B**) allele frequency map of a variation site in the genome, (**C**) PCoA plot, (**D**) phylogenetic tree of all the isolates

PCoA, equivalently multidimensional scaling, is computed as per the R function *cmdscale*, and the phylogenetic tree is constructed using Fitch–Margoliash algorithm (Fitch and Margolia, 1967). The user is provided with an option to group colours based on a known phenotype (e.g. drug susceptibility) or a custom classification. The ability to perform tree computation using external phylogeny package is also supported by saving alignments in a compatible format and visualizing the tree in SVAMP. The PCoA, phylogenetic tree and exporting alignments can be performed on multiple regions of interest within a subset of samples. Integrating popular bam viewers such as *LookSeq* (Manske and Kwiatkowski, 2009) to view read alignment evidence for variants is an added feature of SVAMP.

## 3 RESULTS

We have evaluated the application and scalability of SVAMP using two published datasets: (i) a bacterial population study (Harris *et al.*, 2010) on methicillin-resistant *Staphylococcus aureus* (commonly known as MRSA) and (ii) a worldwide population structure study (Manske *et al.*, 2012) on *Plasmodium falciparum* malaria parasite. Both these example datasets are available for download at http://cbrc.kaust.edu.sa/svamp as a packaged SVAMP bundle.

### 3.1 MRSA outbreak analysis using SVAMP

The MRSA dataset visualised in SVAMP as shown in Figure 1 contains 4310 SNP sites determined from 63 isolates obtained from various hospitals across 15 countries, spanning a period of >25 years. The linear phylogenetic tree constructed using SVAMP is shown in Supplementary Figure S1, and the circular tree in Supplementary Figure S2 is consistent with that described in the paper by Harris *et al.* (2010). Supplementary Figure S3 shows the Portuguese samples on the tree overlaid on the geographical map displaying the year of isolation and location. Supplementary Figure S4 shows the two European isolates DEN907 and TW20 clearly joining the Asian clade. From Supplementary Figure S1, it can also be observed that five isolates from Thailand S21, S24, S39, S42 and S81 obtained from the same hospital cluster together to form a single subclade. Colour coding the isolates based on the country of origin allows the visualization of the geographical map and the tree simultaneously, assisting with making genomic epidemiological inference.

### 3.2 Exploring the population structure of Malaria isolates using SVAMP

The raw sequencing data obtained from *P. falciparum* diversity study (Manske *et al.*, 2012) were mapped using *smalt*, and SNPs were called using *samtools*. Resulting variants were merged using *vcftools*. Only coding region variants that do not fall in *var, rifin* and *stevor* gene (the hypervariable gene families in malaria) sites were included. After filtering for quality and missing data, 26 918 SNPs were retained. This dataset consists of 245 samples from six countries: three from Africa (AFR), two from Southeast Asia (SEA) and Papua New Guinea (PNG). The PCoA analysis using SVAMP in Supplementary Figure S5 clearly shows three different clusters as three different groups AFR, SEA and PNG, as seen in the paper by Manske *et al.* (2012). As expected, individual continental PCoA analyses demonstrate separation between East and West African samples (Supplementary Fig. S6) and between Thailand and Cambodia samples. The commands and parameters used to obtain the final dataset used in SVAMP are explained in the Supplementary Materials.

### 3.3 Memory and computational speed of SVAMP on MRSA and malaria datasets

Memory usage and computational speed of SVAMP was evaluated on a laptop computer with 2 cores (4 GB RAM) and on a workstation with 12 CPU cores (96 GB RAM). The results were averaged for both MRSA and malaria datasets and are shown in Table 1.

**Table 1.** Memory and speed of SVAMP on malaria and MRSA datasets

Dataset (N, K)	Size on disk (MB)	Average RAM usage (GB)	Time to load data (s)	Time to compute PCoA (s)	Time to construct tree
MRSA (63, 4310)	21.3	0.23	4	1	20 s
Malaria (245, 26 918)	637	1.23	50	60	4.7 h

*Note:* N: number of samples; K: number of variants.

## CONCLUSIONS

By using the sequence variant and associated geographical information, we believe the software SVAMP will aid greatly in analysing isolates from an outbreak, as well as predicting the population structure in epidemiological studies.

*Funding*: KAUST faculty baseline funding to A.P.; Medical Research Council (UK) grant MR/J005398/1 to T.G.C. and M.D.P.

*Conflict of Interest*: none declared.

## Supplementary Material

Supplementary Data
