# Supplementary material for: SVAMP: sequence variation analysis, maps and phylogeny
Source: Bioinformatics. 2014 Apr 3;30(15):2227–9. doi: 10.1093/bioinformatics/btu176 (PMC4103593; doi:10.1093/bioinformatics/btu176)
Supplement: Supplementary Data [file supp_30_15_2227__index.html]

SVAMP: Sequence Variation Analysis, Maps and Phylogeny. — SVAMP: sequence variation analysis, maps and phylogeny — SVAMP: sequence variation analysis, maps and phylogeny — Supplementary Data 

# SVAMP: sequence variation analysis, maps and phylogeny

## Supplementary Data

files

**Files in this Data Supplement:**

- Supplementary Data - docx file
