# Supplementary material for: SVAMP: sequence variation analysis, maps and phylogeny
Source: Bioinformatics. 2014 Apr 3;30(15):2227–9. doi: 10.1093/bioinformatics/btu176 (PMC4103593; doi:10.1093/bioinformatics/btu176)
Supplement: Supplementary Data [file supp_btu176_SVAMP_supplementary_V7.docx]

**Supplementary Material**

SVAMP: Sequence Variation Analysis, Maps and Phylogeny.

Raeece Naeem^1^, Lailatul Hidayah^1^, Mark D. Preston^2^, Taane G. Clark^2^ and Arnab Pain^1^

^1^ Pathogen Genomics Laboratory, Computational Bioscience Research Center, King Abdullah University of Science and Technology (KAUST), Thuwal-23955-6900, Kingdom of Saudi Arabia

^2^ Department of Pathogen Molecular Biology, London School of Hygiene and Tropical Medicine, London WC1E 7HT, United Kingdom.

**Supplemental Figures**


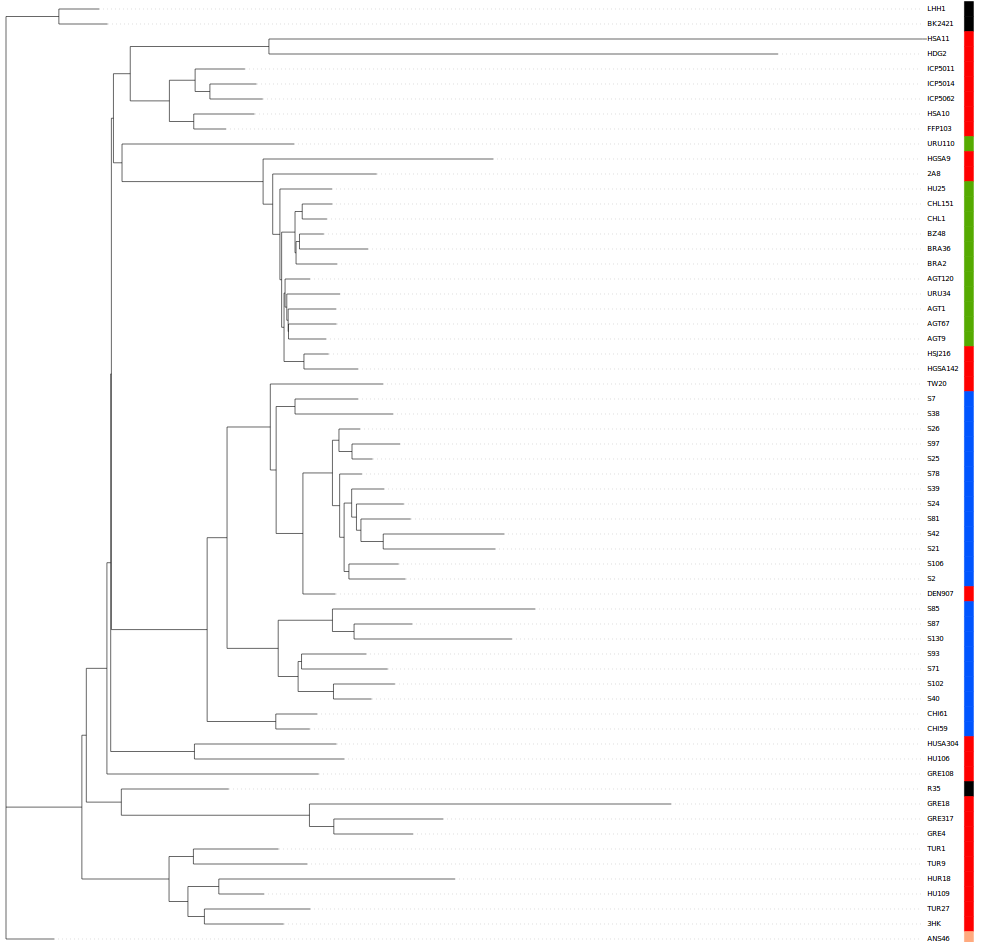


**Figure S1**: Linear phylogenetic tree constructed using SVAMP shows 63 isolates of MRSA color-coded according to the continents of isolation. The continental origin of each isolate is indicated by the color of the isolate name: blue, Asia; black, North America; green, South America; red, Europe; and yellow, Australasia. ([Harris, et al., 2010](#_ENREF_3)). The 5 Thai isolates S21, S24, S39, S42 and S81 obtained from the same hospital cluster together in a single sub clade.


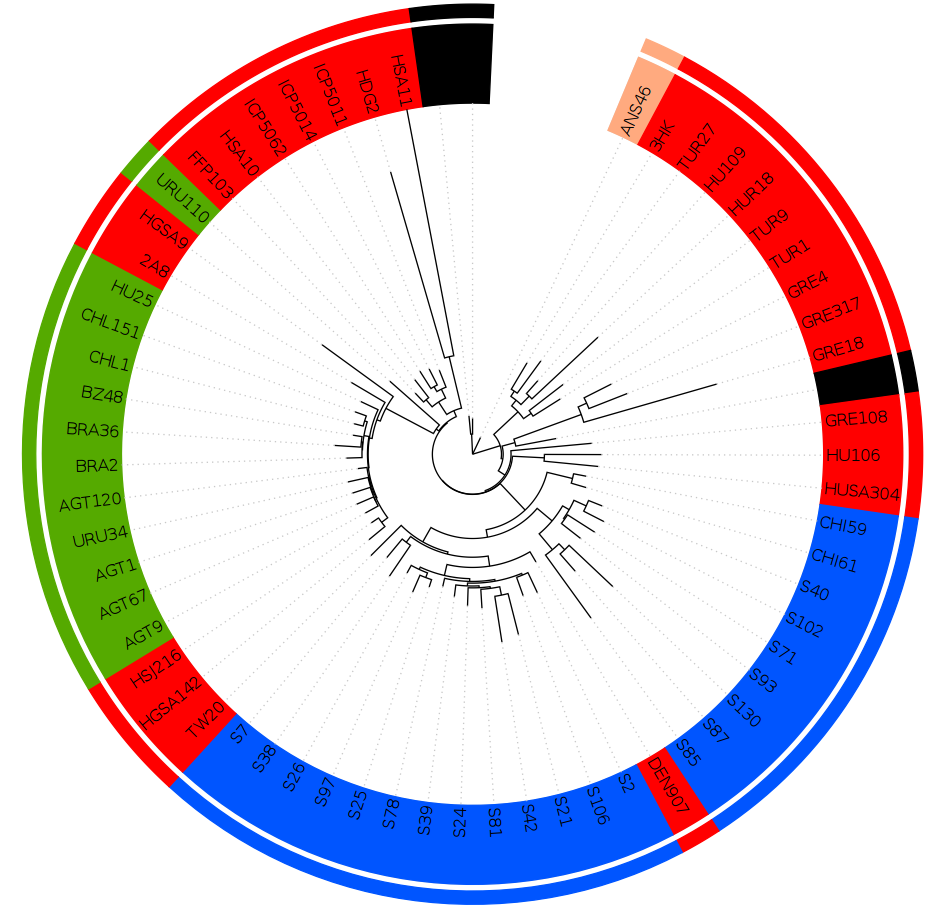


**Figure S2**: Circular phylogenetic tree constructed using SVAMP shows 63 isolates of MRSA color-coded according to the continents of isolation.


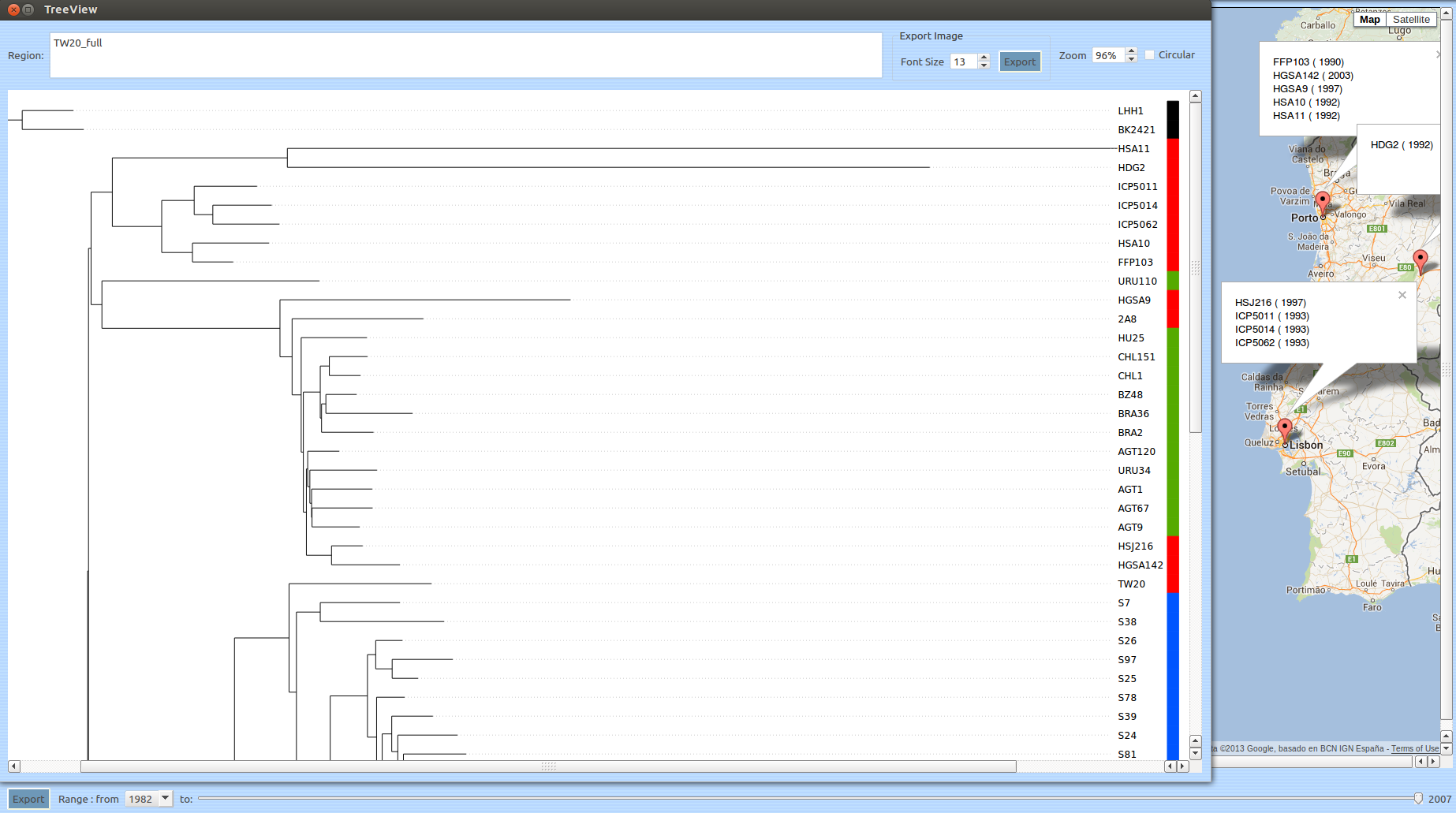


**FigureS3**: All seven Portuguese isolates (HSA11, HDG2, ICP5011, ICP5014, ICP5062, HSA10, FFFP103) recovered between 1990 and 1993 clustered together, whereas the three Brazilian clone isolates(HGSA9, HSJ216, HGSA142) clustered within the South American clade, strongly supporting the hypothesis that this second wave in Portugal resulted from the introduction of a South American variant.([Harris, et al., 2010](#_ENREF_3)).


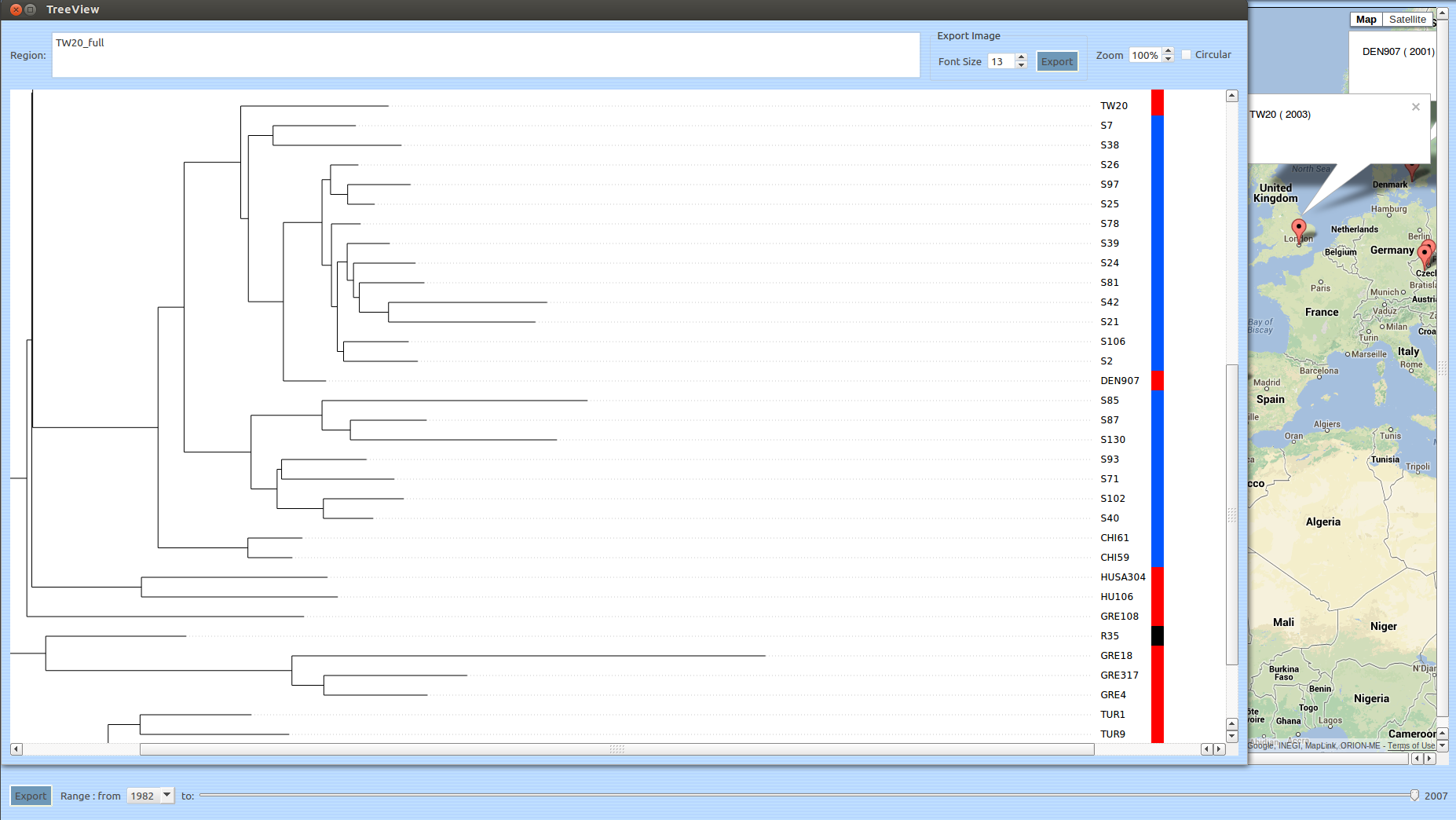


**FigureS4**: Two European isolates DEN907 and TW20 clearly joining the Asian clade. ([Harris, et al., 2010](#_ENREF_3)).


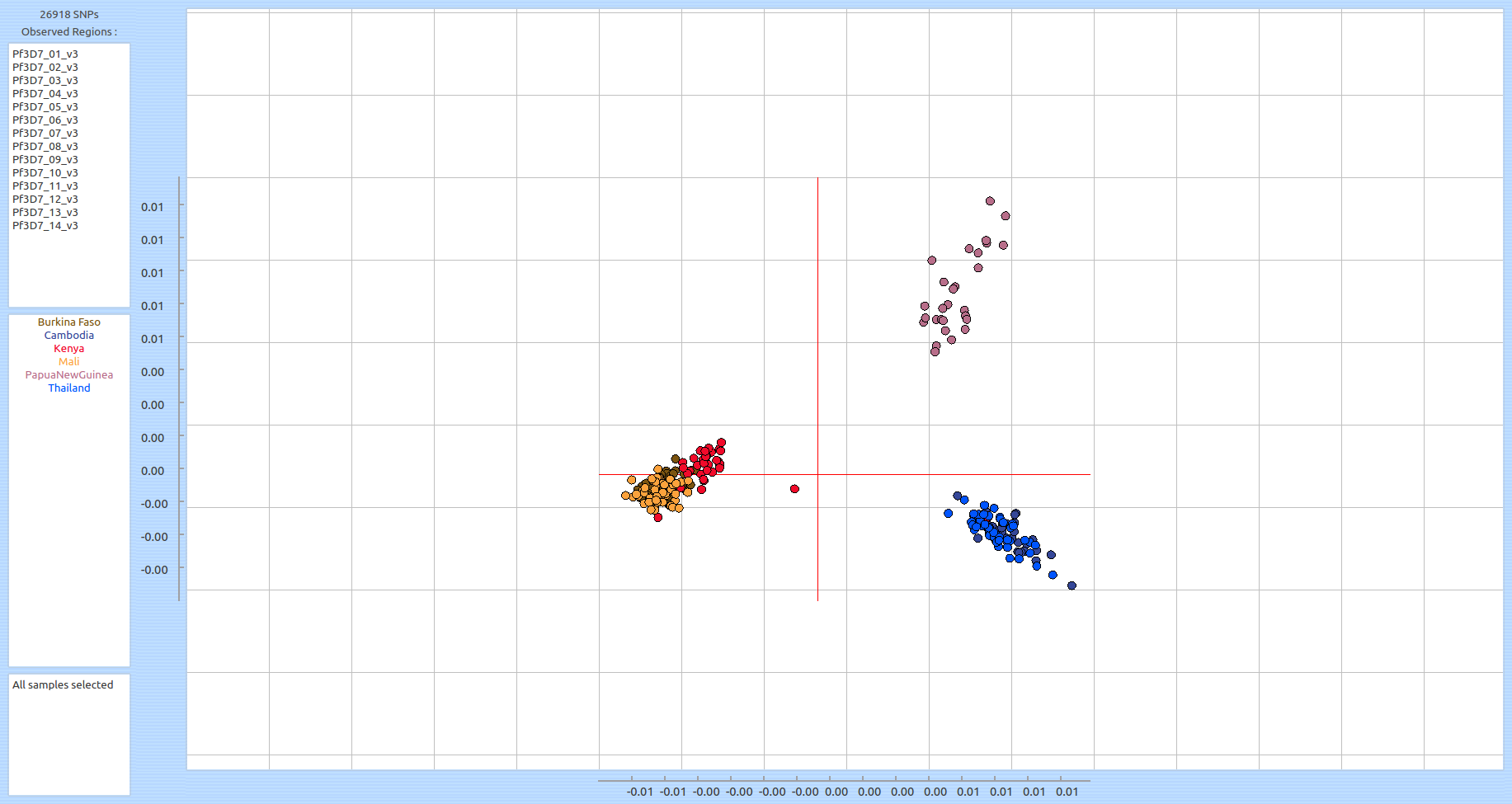


**FigureS5**: PCoA plot from SVAMP on the malaria dataset shows formation of 3 cluster groups: Africa (AFR), South East Asia (SEA) and Papua New Guinea (PNG) as previously shown in ([Manske, et al., 2012](#_ENREF_5)).


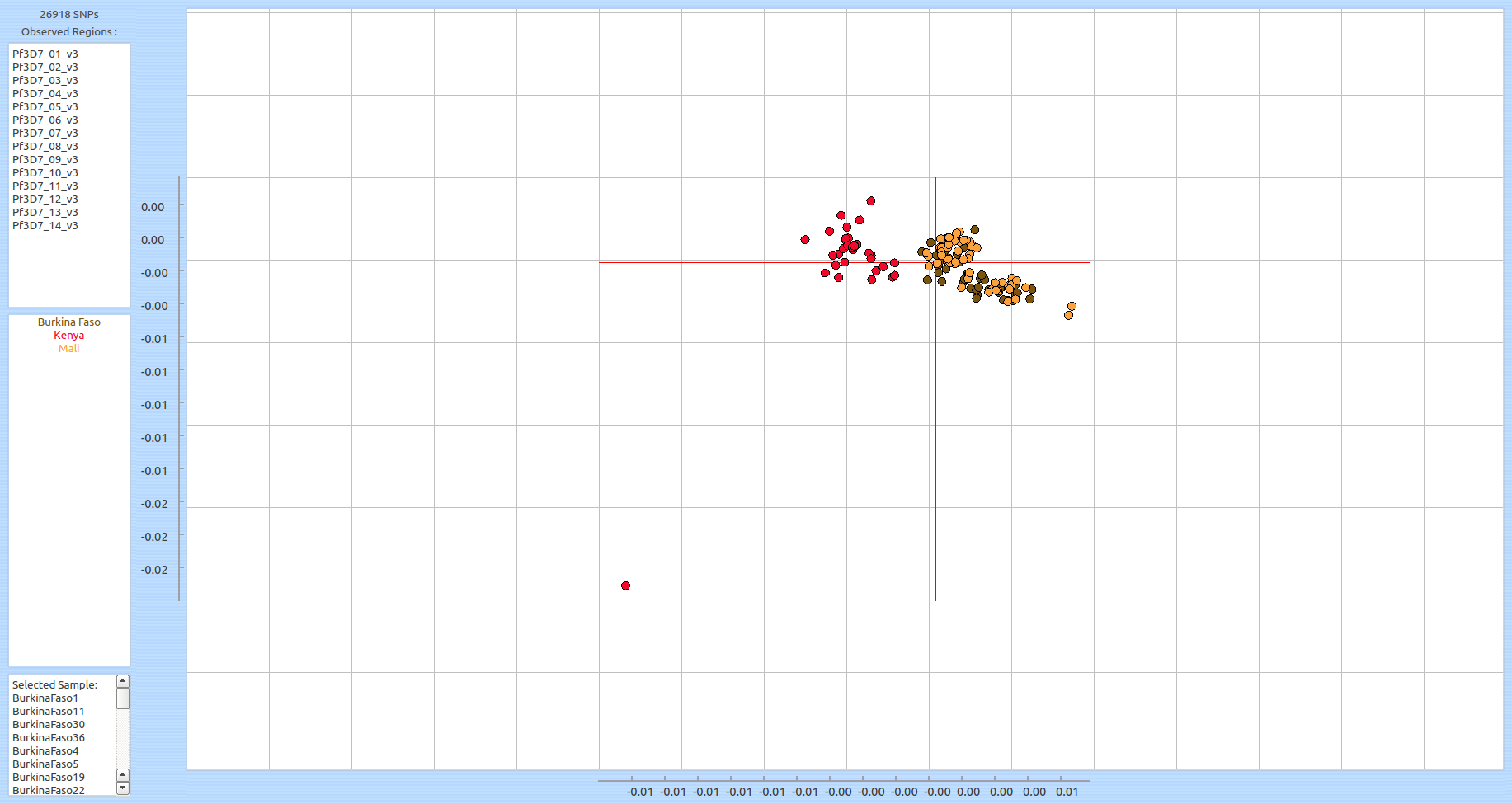


**FigureS6**: PCoA plot from SVAMP on African isolates shows the separation of two cluster groups East Africa and West Africa (as shown in ([Manske, et al., 2012](#_ENREF_5))).


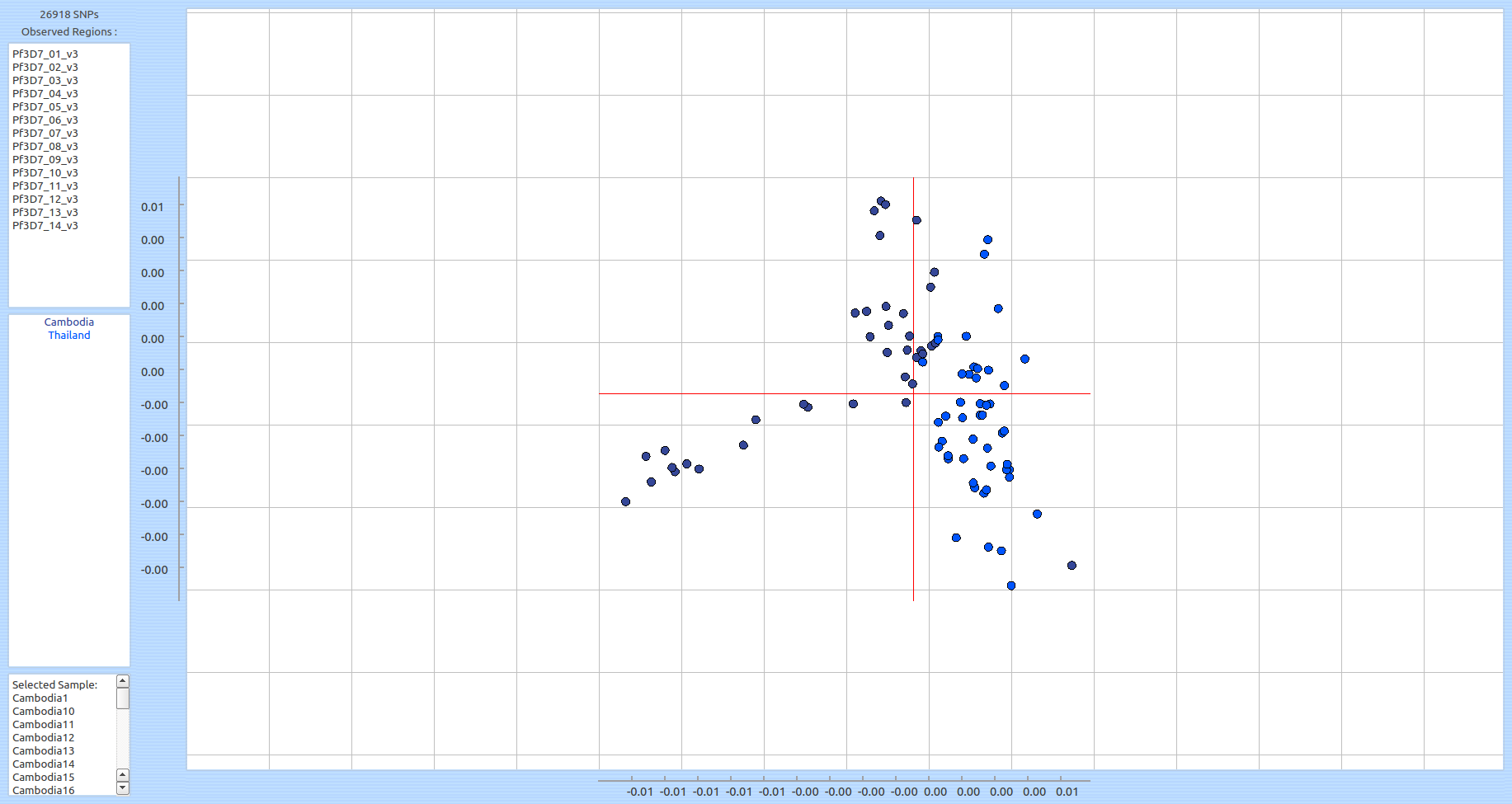


**FigureS7**: PCoA plot from SVAMP on South East Asia isolates shows separation of isolates from Thailand and Cambodia.

#
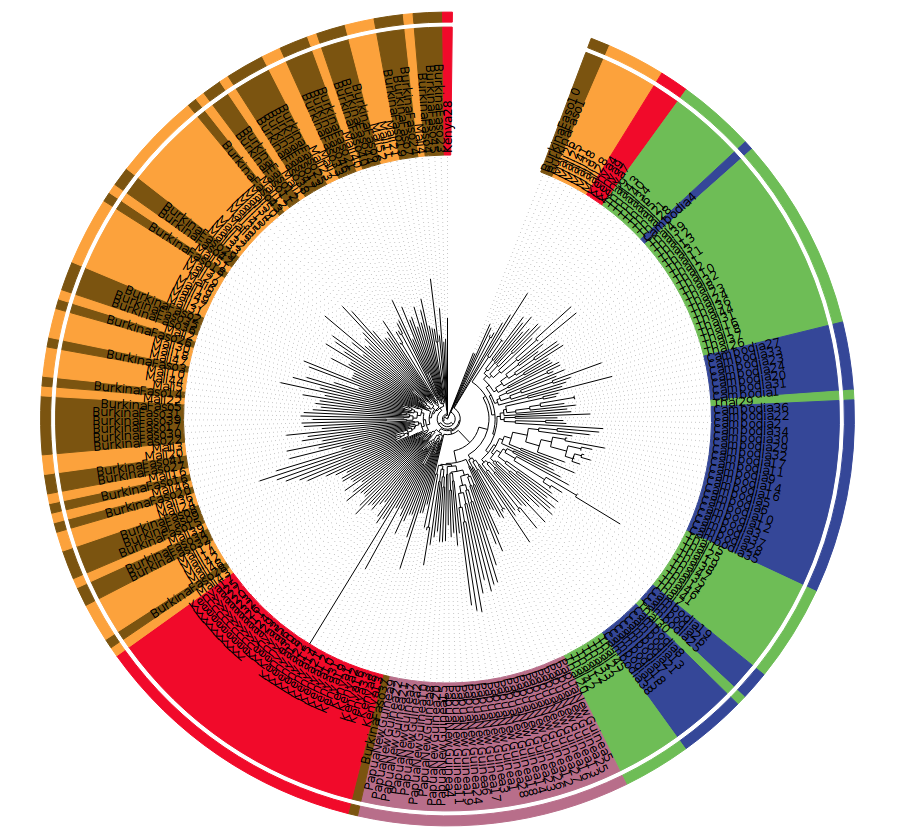


**FigureS8**: Maximum likelihood tree generated using RAxML ([Stamatakis, 2006](#_ENREF_7)) package on malaria dataset and visualized on SVAMP

#
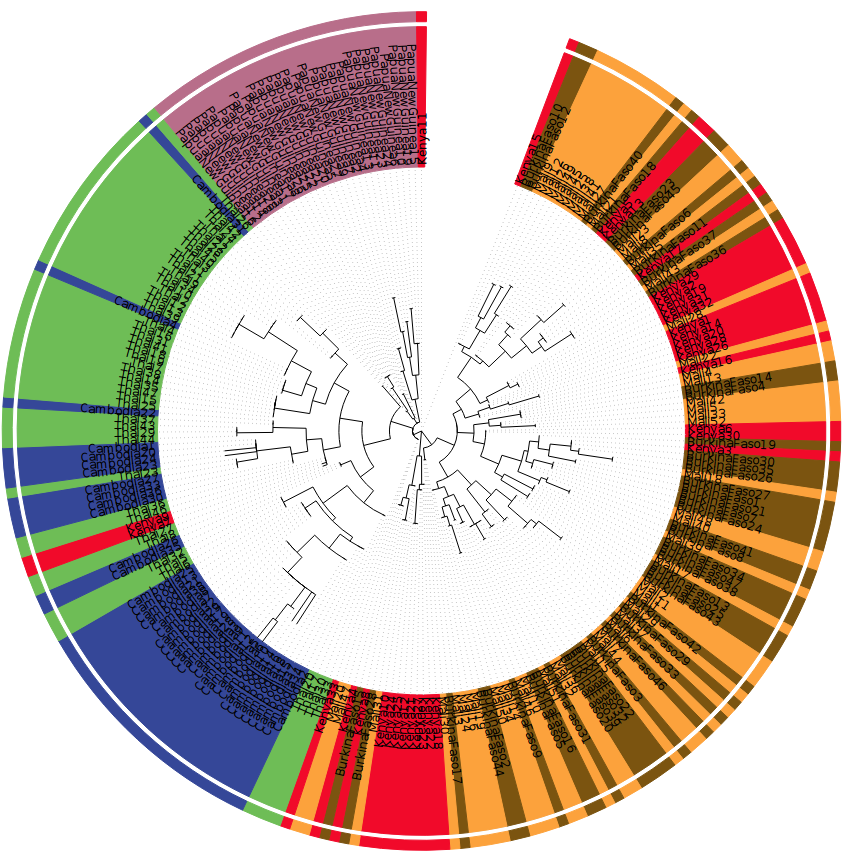


**FigureS9**: Phylogenetic tree generated using BEAST ([Drummond, et al., 2012](#_ENREF_2)) package on malaria dataset and visualized on SVAMP

# Supplemental Methods

## MRSA data set preparation.

MRSA reference, annotation GFF file, 4310 core SNPs and the geographical metadata of isolates were downloaded from ([Harris, et al., 2010](#_ENREF_3)). The SNP table was converted to VCF using a script and the metadata prepared using SVAMP data preparation script *prepare_svamp_bundle.pl*. The resulting VCF, FASTA, GFF and db files are bundled together and are available for download from the SVAMP website.

## Malaria data set preparation.

Raw Sequencing data of *P. falciparum* isolates were obtained from study ([Manske, et al., 2012](#_ENREF_5)). The reads were mapped to *P. falciparum* 3D7 reference v3.0 using *smalt* ([www.sanger.ac.uk/smalt](http://www.sanger.ac.uk/smalt)) and single sample VCF files were obtained using samtools ([Li, et al., 2009](#_ENREF_4)) command

samtools mpileup -uf Pf3D7_v3.fasta sample.bam | bcftools view -cg - > sample_full.vcf

The resulting single sample VCF files were concatenated using VCFtools ([Danecek, et al., 2011](#_ENREF_1)) to produce a multi-sample VCF file containing 245 isolates and 2.6 million variants. Only variants that fall in coding regions excluding *var, rifin* and *stevor* genes were extracted using *bedtools* ([Quinlan and Hall, 2010](#_ENREF_6))

Quality filtering on the resulting variants was performed using the command

Vcf-annotate –filter Q=70/d=100 variants.vcf > typablevariants.vcf

This resulted in 504,917 variants. The variants that had no missing genotypes were obtained using the command

Vcftools –geno 1.0 typablevariants.vcf –recode –out final

The SVAMP bundle with VCF containing 26918 variant sites, reference fasta, gff and db file are available for download from the SVAMP website.

# References

Danecek, P.*, et al.* (2011) The variant call format and VCFtools, *Bioinformatics*, **27**, 2156-2158.

Drummond, A.J.*, et al.* (2012) Bayesian Phylogenetics with BEAUti and the BEAST 1.7, *Mol Biol Evol*, **29**, 1969-1973.

Harris, S.R.*, et al.* (2010) Evolution of MRSA during hospital transmission and intercontinental spread, *Science*, **327**, 469-474.

Li, H.*, et al.* (2009) The Sequence Alignment/Map format and SAMtools, *Bioinformatics*, **25**, 2078-2079.

Manske, M.*, et al.* (2012) Analysis of Plasmodium falciparum diversity in natural infections by deep sequencing, *Nature*, **487**, 375-379.

Quinlan, A.R. and Hall, I.M. (2010) BEDTools: a flexible suite of utilities for comparing genomic features, *Bioinformatics*, **26**, 841-842.

Stamatakis, A. (2006) RAxML-VI-HPC: Maximum likelihood-based phylogenetic analyses with thousands of taxa and mixed models, *Bioinformatics*, **22**, 2688-2690.
